# Supplementary material for: Structural and Thermal Characterization of Protein Isolates from Australian Lupin Varieties as Affected by Processing Conditions
Source: Foods. 2023 Feb 21;12(5):908. doi: 10.3390/foods12050908 (PMC10001375; doi:10.3390/foods12050908)
Supplement: Supplementary file 1 [file foods-12-00908-s001.zip › foods-2112642-supplementary.pdf]

Structural and Thermal Characterization of Protein Isolates from Australian Lupin Varieties as Affected by Processing Conditions

Lavaraj Devkota [1], Konstantina Kyriakopoulou [2], Robert Bergia [2] and Sushil Dhital [1,*]

[1] Department of Chemical and Biological Engineering, Monash University, Clayton, VIC 3800, Australia

[2] Archer-Daniels-Midland (ADM), James R. Randall Research Centre, Decatur, IL 62521, USA

* **Correspondence:** sushil.dhital@monash.edu; Tel.: +61-3-9905-9344

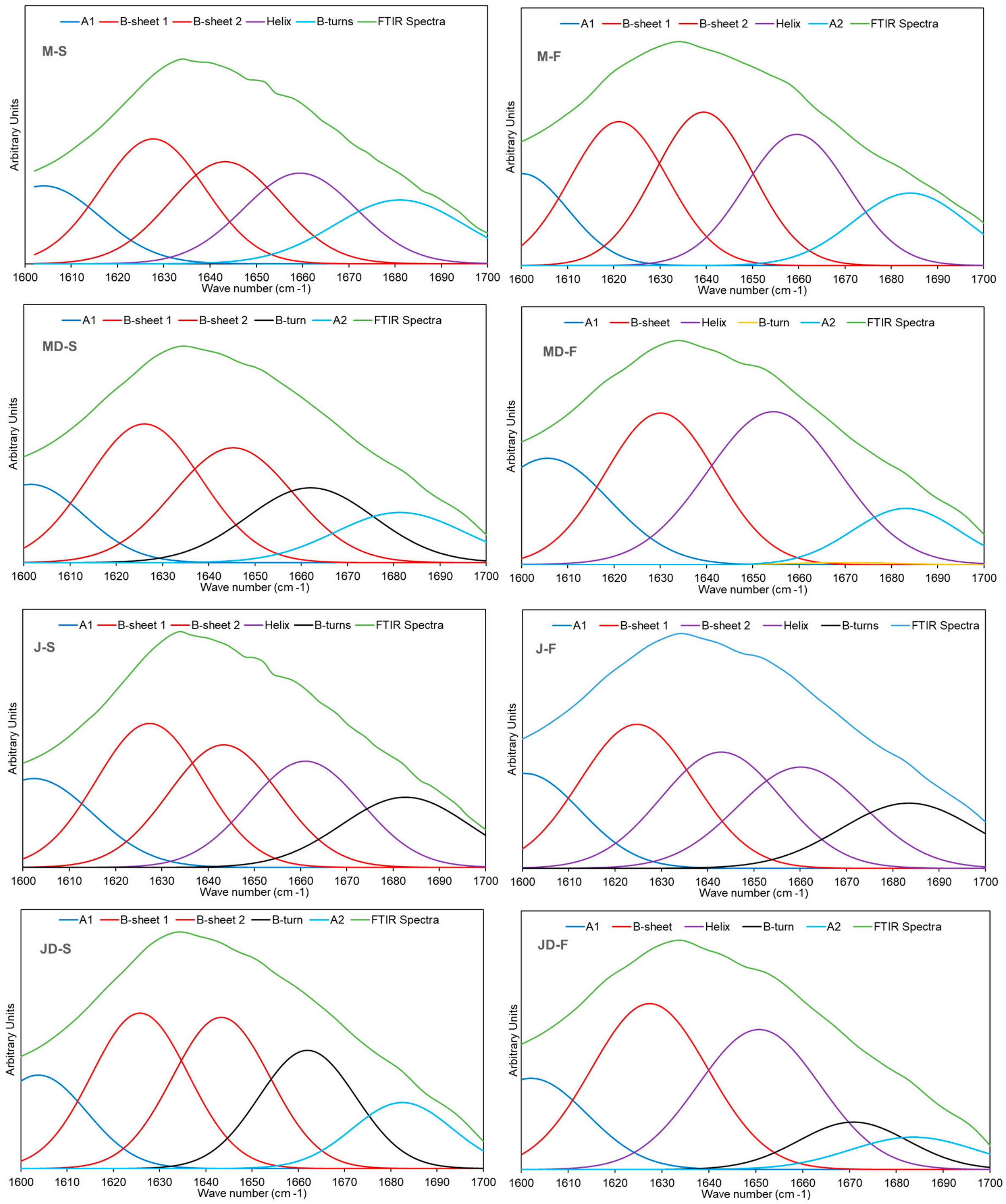
A1
B-sheet 1
B-sheet 2
Helix
B-turns
FITR Spectra
M-S
Arbitrary Units
Wave number (cm -1)
A1
B-sheet 1
B-sheet 2
Helix
A2
FTIR Spectra
M-F
Arbitrary Units
Wave number (cm -1)
A1
B-sheet 1
B-sheet 2
B-turn
A2
FTIR Spectra
MD-S
Arbitrary Units
Wave number (cm -1)
A1
B-sheet
Helix
B-turn
A2
FTIR Spectra
MD-F
Arbitrary Units
Wave number (cm -1)
A1
B-sheet 1
B-sheet 2
Helix
B-turns
FTIR Spectra
J-S
Arbitrary Units
Wave number (cm -1)
A1
B-sheet 1
B-sheet 2
Helix
B-turns
FTIR Spectra
J-F
Arbitrary Units
Wave number (cm -1)
A1
B-sheet 1
B-sheet 2
B-turn
A2
FTIR Spectra
JD-S
Arbitrary Units
Wave number (cm -1)
A1
B-sheet
Helix
B-turn
A2
FTIR Spectra
JD-F
Arbitrary Units
Wave number (cm -1)
1600 1610 1620 1630 1640 1650 1660 1670 1680 1690 1700

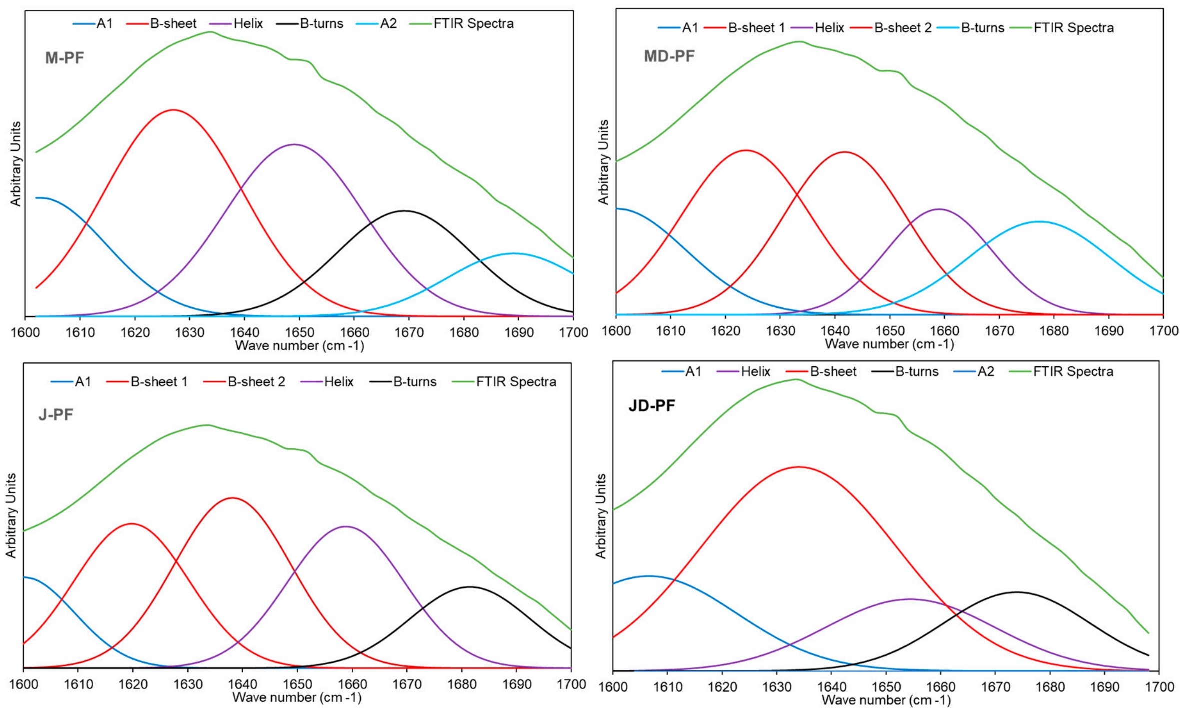

Figure S1. *Amide 1 region of the FTIR curve and protein secondary structure quantification using Gaussian curve fitting.* MD-F=Defatted Murringo direct freeze dried, JD-F=Defatted Jurien direct freeze dried, M-F=Full fat Murringo direct freeze dried, J-F=Full fat Jurien direct freeze dried, MD-PF=Defatted Murringo pasteurized and freeze dried, JD-PF=Defatted Jurien pasteurized and freeze dried, M-PF=Full fat Murringo pasteurized and freeze dried, J-PF=Full fat Jurien pasteurized and freeze dried, MD-S=Defatted Murringo spray dried, JD-S=Defatted Jurien spray dried, M-S=Full fat Murringo spray dried, J-S=Full fat Jurien spray dried.

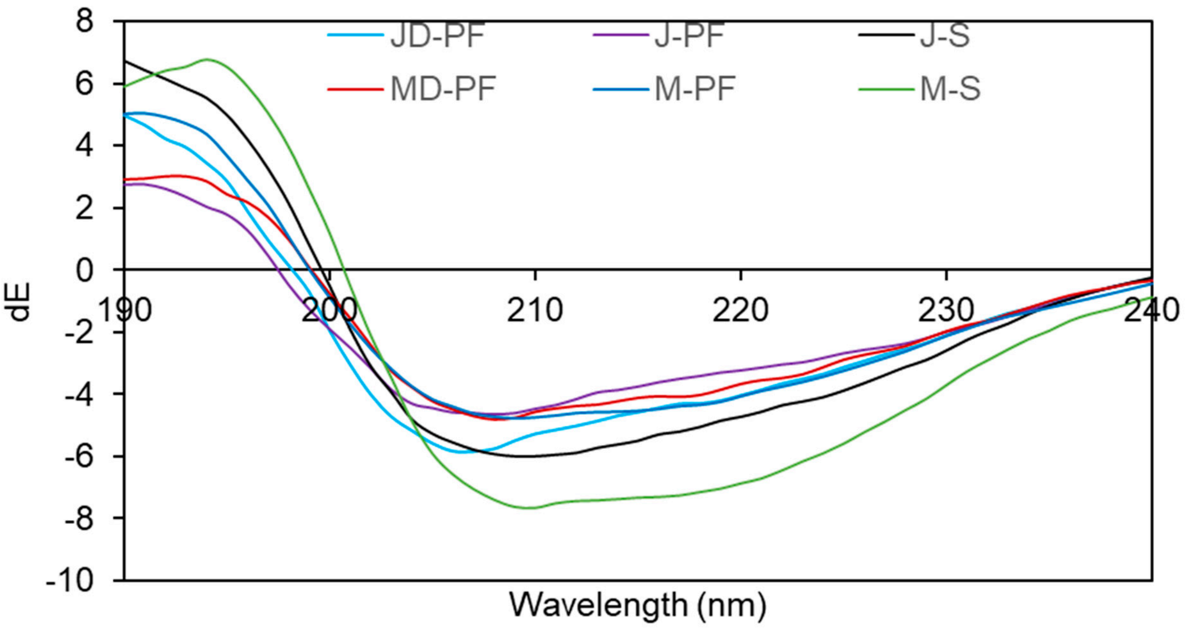

Figure S2. representative *f*ar UV *c*ircular dichroism curve for lupin protein isolates *obtained by different processing techniques.* MD-PF=Defatted Murringo pasteurized and freeze dried, JD-PF=Defatted Jurien pasteurized and freeze dried, M-PF=Full fat Murringo pasteurized and freeze dried, J-PF=Full fat Jurien pasteurized and freeze dried, M-S=Full fat Murringo spray dried, J-S=Full fat Jurien spray dried.
